# Comorbid Arthritis Is Associated With Lower Health-Related Quality of Life in Older Adults With Other Chronic Conditions, United States, 2013–2014

**DOI:** 10.5888/pcd14.160495

**Published:** 2017-07-27

**Authors:** Eric Havens, S. Lane Slabaugh, Charles G. Helmick, Tristan Cordier, Matthew Zack, Vipin Gopal, Todd Prewitt

**Affiliations:** 1 Humana Inc, Louisville, Kentucky; 2 Arthritis Program, Division of Population Health, Centers for Disease Control and Prevention, Atlanta, Georgia

## Abstract

**Introduction:**

Arthritis is related to poor health-related quality of life (HRQoL) in adults aged 18 years or older. We sought to determine whether this relationship persisted in an older population using claims-based arthritis diagnoses and whether people who also had arthritis and at least 1 of 5 other chronic conditions had lower HRQoL.

**Methods:**

We identified adults aged 65 years or older with Medicare Advantage coverage in November or December 2014 who responded to an HRQoL survey (Healthy Days). For respondents with and without arthritis, we used linear regression to compare mean physically, mentally, and total unhealthy days, overall and in 5 comorbidity subgroups (coronary artery disease, congestive heart failure, chronic obstructive pulmonary disease, diabetes, and hypertension), accounting for age, sex, dual Medicaid/Medicare eligibility, rural/urban commuting area, and Charlson Comorbidity Index.

**Results:**

Of the 58,975 survey respondents, 44% had arthritis diagnosed through claims. Respondents with arthritis reported significantly more adjusted mean physically, mentally, and total unhealthy days than those without arthritis (*P* < .001). Older adults with arthritis and either congestive heart failure, chronic obstructive pulmonary disease, diabetes, or hypertension reported significantly more adjusted physically, mentally, and total unhealthy days than older adults without arthritis but with the same chronic conditions.

**Conclusions:**

In older adults, having arthritis is associated with lower HRQoL and even lower HRQoL among those with at least 1 of 5 other common chronic conditions. Because arthritis is so common among older adults, improving HRQoL depends on managing both underlying chronic conditions and any accompanying arthritis.

## Introduction

Arthritis, including rheumatic conditions, is a highly prevalent (29%) chronic condition in Medicare beneficiaries and is a leading cause of disability (1–3). Almost half of people with heart disease or diabetes and one-third of those with obesity have arthritis (4,5). With such conditions, arthritis increases serious psychological distress and restricts social participation (2,6).

Arthritis is associated with diminished health-related quality of life (HRQoL) in several studies of adults aged 18 years or older, many of which used the Centers for Disease Control and Prevention (CDC) Healthy Days (or HRQoL-4) instrument (7–12). This instrument contains 4 questions that ask about a person’s perceived physical and mental health (Table 1) (13,14). Healthy Days is a valid and reliable measure of HRQoL, is highly correlated with objective health measures (15–19), and has been incorporated into national, self-reported surveys, including the Behavioral Risk Factor Surveillance System and the National Health and Nutrition Examination Survey (13,19–21).

**Table 1:** Centers for Disease Control and Prevention Healthy Days Instrument^a^

Number	Question
1	Would you say that in general your health is excellent, very good, good, fair, or poor?
2	Now thinking about your physical health, which includes physical illness and injury, for how many days during the past 30 days was your physical health not good?
3	Now thinking about your mental health, which includes stress, depression, and problems with emotions, for how many days during the past 30 days was your mental health not good?
4	During the past 30 days, for about how many days did poor physical or mental health keep you from doing your usual activities, such as self-care, work, or recreation?

a Also called the HRQoL-4 survey and includes all 4 questions.

The association of arthritis with lower HRQoL could vary by the age. We combined administrative claims diagnoses of older adults with Healthy Days survey data to 1) characterize the relationship between arthritis and HRQoL in older adults and 2) assess the impact of comorbid arthritis on HRQoL among those with coronary artery disease, congestive heart failure, chronic obstructive pulmonary disease, diabetes, or hypertension.

## Methods

### Source population and general survey

Humana Inc., a health and well-being company that serves millions of people across the country through Medicare Advantage, stand-alone Prescription Drug Plan, and commercial plan offerings, selected the Healthy Days quality of life instrument to measure progress toward a goal of improving the health of the communities it serves by 20% by the year 2020 (22,23). A stratified, random sample of health plan participants across several plans received a voice-activated technology (VAT) telephone survey using the 4 Healthy Days questions. This cross-sectional survey was administered across multiple medical coverage plans from November 24 through December 24, 2014. Survey results were linked to administrative medical claims and enrollment data by a unique patient identifier. Details of the survey methods have been published elsewhere (24).

The random survey sample was stratified to ensure that the geographic locations (25 geographies based on county codes), medical coverage plans (Medicare, employer group, and individual; ultimately only the Medicare population was eligible for this study), and chronic conditions in the survey sample population were representative of the larger plan population. Only individuals with a viable telephone number who responded within 3 consecutive calls and answered both questions on physically and mentally unhealthy days were included in the survey sample. Of the 4.1 million eligible medical plan enrollees, 870,791 were called. Of those, 618,984 were unreachable by telephone, and 118,034 did not answer both questions on Healthy Days, leaving a stratified random sample of 133,773 individuals (15%) with complete Healthy Days responses (Appendix A).

Survey weighting was used to standardize the sample respondents to the entire plan population as of September 30, 2014. An iterative proportional fitting (ranking) algorithm was performed to account for differences between respondents and nonrespondents in sex, age, and 5 diagnosed conditions (coronary artery disease, congestive heart failure, chronic obstructive pulmonary disease, diabetes, and hypertension). The ranking algorithm calculates weights one factor at a time, cycling through the desired demographics repeatedly until convergence is reached at some final weight, allowing for empty data cells to be accounted for (25,26).

### Study sample selection

We defined the study reporting period as the 12 months before the survey date for each respondent, encompassing November 24, 2013, through December 24, 2014. From the stratified, random sample of 133,773 survey respondents, individuals were selected for this study if they met the following criteria: 1) being aged 65 years or older at the start of the observation period, 2) being actively enrolled in a Medicare Advantage plan at the time of the survey, and 3) having had continuous Medicare Advantage enrollment during the observation period. A total of 58,975 individuals (44%) met these criteria (Appendix A). We focused on Medicare Advantage enrollees so that we could investigate a population with sufficient size, prevalence of arthritis and other chronic conditions, and homogeneity. The study was limited to individuals aged 65 years or older because people younger than 65 have extended disability, end-stage renal disease, or other eligibility criteria known to be substantially associated with Healthy Days.

### Measurements and analyses

Arthritis was defined as at least 1 inpatient claim or 2 outpatient or physician office claims during the 12-month reporting period that included *International Classification of Diseases, Ninth Revision, Clinical Modification* (ICD-9-CM) codes related to arthritis as developed by the National Arthritis Data Workgroup (Table 2). Using ICD-9-CM codes and clinical judgment, one author (T.P.) categorized arthritis as inflammatory or noninflammatory on the basis of the following convention: if arthritis was most probably related to a structural deformity or wear from anatomical causes, it was listed as noninflammatory; if there was any chance the arthritis could be related to inflammation, it was listed as inflammatory. Healthy Days and activity limitation responses were extracted from the VAT survey. Physically and mentally unhealthy days were calculated individually and were summed to yield a total number of unhealthy days, capped at 30 days, per CDC methodology, which is supported by actual survey response patterns that indicate most individuals report either physically or mentally unhealthy days and a very small percentage report equal numbers for both (13,14).

**Table 2:** Diagnostic Codes for Arthritis and Other Rheumatic Conditions as Developed by the National Arthritis Data Workgroup

ICD-9-CM Code^a^	Inflammatory Arthritis^b^	Disease or Condition
710	Yes	Diffuse diseases of connective tissue
711	Yes	Infectious arthropathies
712	Yes	Crystal arthropathies
713		Arthropathy associated with other disorders classified elsewhere
714	Yes	Rheumatoid arthritis/inflammatory arthropathies
715		Osteoarthritis and allied disorders
716	Yes	Other/unspecified arthropathies (eg, allergic arthritis, transient arthropathy)
719^c^		Other/unspecified joint disorders (eg, joint effusion, walking difficulty)
720	Yes	Ankylosing spondylitis/inflammatory spondylopathies
721	Yes	Spondylosis and allied disorders
725	Yes	Polymyalgia rheumatica
726	Yes	Peripheral enthesopathies and allied conditions
727	Yes	Other disorders of synovium/tendon/bursa (eg, tenosynovitis, synovial cyst)
728^d^		Disorders of muscle/ligament/fascia
729.0	Yes	Rheumatism, unspecified/fibrositis
729.1	Yes	Myalgia/myositis unspecified
729.4	Yes	Fasciitis, unspecified
095.6	Yes	Syphilis of muscle
095.7	Yes	Syphilis of synovium/tendon/bursa
098.5	Yes	Gonococcal infection of joint
099.3	Yes	Reiter’s disease
136.1	Yes	Behcet’s syndrome
274	Yes	Gout
277.2	Yes	Other disorders of purine/pyrimidine metabolism disorder
287.0	Yes	Allergic purpura
344.6		Cauda equina syndrome
353.0		Brachial plexus lesion/thoracic outlet syndromes
354.0		Carpal tunnel syndrome
355.5		Tarsal tunnel syndrome
357.1	Yes	Polyneuropathy, collagen vascular disease
390	Yes	Rheumatic fever, no heart involvement
391	Yes	Rheumatic fever, heart involvement
437.4	Yes	Cerebral arteritis
443.0	Yes	Raynaud’s syndrome
446	Yes	Polyarteritis nodosa and allied conditions
447.6	Yes	Arteritis, unspecified
696.0	Yes	Psoriatic arthropathy

Abbreviation: ICD-9-CM, International Classification of Diseases, Ninth Revision, Clinical Modification.

a Use of 3-digit codes is intended to include all subdivided 4- and 5-digit codes, and the use of 4-digit codes is intended to include all subdivided 5-digit codes.

b Inflammatory arthritis was defined on the basis of professional judgment as any arthritis that could be related to an inflammatory process. The final list was cross-referenced with and corresponded well to information found at http://www.arthritis.org/about-arthritis/types/.

c Excludes ICD-9-CM code 719.1.

d Excludes ICD-9-CM codes 728.4 and 728.5.

We compared individuals with and without arthritis with respect to their mean physically, mentally, and total unhealthy days and days with activity limitation, both overall and for the effect of arthritis on these measures for those with 1 of 5 chronic conditions (coronary artery disease, congestive heart failure, chronic obstructive pulmonary disease, diabetes, or hypertension). These chronic conditions were selected, because they are common in an older adult population and strongly affect overall population health. Conditions were identified during the observation period by using corresponding ICD-9-CM codes (Appendix B) documented on at least 1 inpatient or 2 outpatient or physician office visit claims.

We used linear regression, adjusted for age, sex, dual Medicaid/Medicare eligibility status, rural/urban commuting area, and the Charlson Comorbidity Index (CCI) to account for potential confounders affecting the relationship between arthritis and unhealthy days. We did not include race/ethnicity because of lack of reliable data (27). Dual Medicare/Medicaid eligibility means that the patient qualifies both for Medicare (due to age or disability status) and Medicaid (based on income level). Rural/urban commuting area codes are used to define 4 living areas (urban, suburban, large rural and small rural) based on zip codes. The CCI identifies various coexisting diseases from diagnosis codes in administrative data and quantifies total comorbidity by summing values of each disease (1, 2, 3, or 6) depending on its associated risk of death (low score ≤3, high score ≥13) (28). To avoid double counting, ICD-9-CM codes related to arthritis were excluded from the CCI calculation for all analyses. For analyses of specific chronic conditions, we used separate CCI definitions that excluded ICD-9-CM codes related to the condition of interest.

The analysis was weighted by using the survey sample weights, and all analyses were completed using SAS Enterprise Guide 5.4 (SAS Institute, Inc.), with an a priori α set at .05. This retrospective study was conducted as part of Humana’s normal quality improvement operations, did not meet the US Department of Health and Human Services’ regulatory definition of research under 45 Code of Federal Regulations 46.102(d), and thus did not require informed consent or institutional review board approval.

## Results

The final study sample consisted of 58,975 survey respondents after excluding 74,798 respondents who did not meet study inclusion criteria (10,539 younger than age 65 years; 50,041 not enrolled in a Medicare Advantage plan; and 14,218 without 12 months continuous enrollment) (Appendix A). The prevalence of arthritis in this study sample was 44% (n = 25,724) with 50% (n = 12,977) categorized as inflammatory and 53% (n = 13,583) as noninflammatory arthritis (some patients had both). Compared with individuals without arthritis (n = 33,251), individuals with arthritis were older, more likely to be women and dually eligible for Medicare and Medicaid, used preferred provider organizations, and had more comorbidities (ie, higher CCI) (all *P* < .001), although many of these differences were small and, given the large sample sizes, may not have been clinically meaningful (Table 3).

**Table 3:** Characteristics of Survey Respondents Aged 65 Years or Older With Medicare Advantage Coverage in November or December 2014 and Having 12 Months Continuous Enrollment Before Survey Date (N = 58,975), by Arthritis Status

Characteristic	With Arthritis (n = 25,724)	Without Arthritis (n = 33,251)	*P* Value^a^
**Age, mean (SE)**	75.3 (0.07)	74.6 (0.06)	<.001
**Charlson Comorbidity Index^b^, mean (SE)**	7.6 (0.03)	6.6 (0.03)	<.001
**Female sex, n (%)**	16,969 (61.8)	19,420 (52.6)	<.001
**Dual Medicare/Medicaid eligibility status, n (%)**	2,088 (7.6)	2,245 (6.1)	<.001
**Rural/urban commuting area^c^ n (%)**			
Urban core	18,760 (68.4)	24,796 (67.2)	.32
Suburban	3,344 (16.8)	6,640 (18.0)	
Large rural	749 (7.5)	2,840 (7.7)	
Small rural	671 (6.9)	2,454 (6.6)	
Unknown	35 (0.5)	176 (0.5)	

Abbreviation: SE, standard error.

a Significant differences between arthritis and no arthritis groups were determined by using χ^2^ test.

b The Charlson Comorbidity Index identifies various coexisting diseases from diagnosis codes in administrative data and quantifies total comorbidity by summing values of each disease (1, 2, 3, or 6), depending on its associated risk of death (low score ≤3, high score ≥13) (28).

c Rural urban commuting areas define 4 living areas (urban, suburban, large rural, and small rural) based on zip codes.

**Figure 1:**
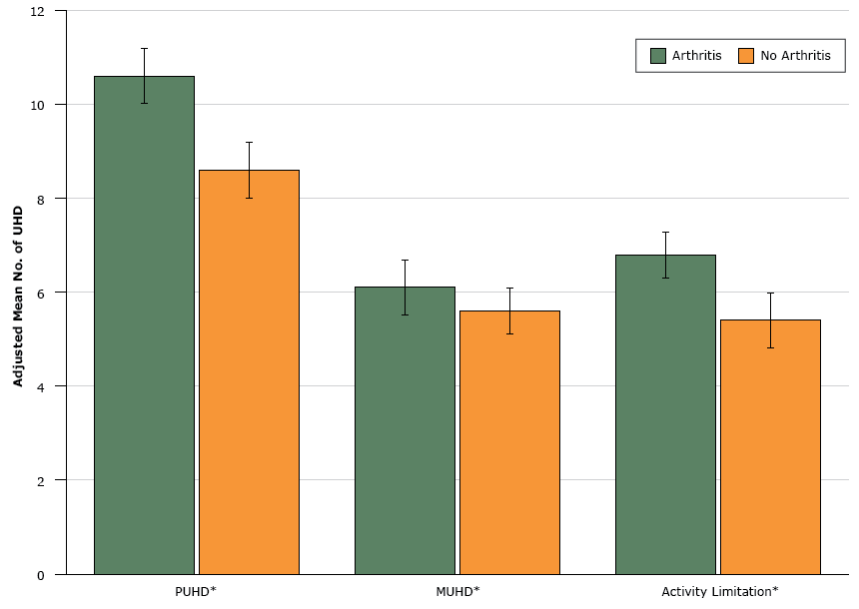
Adjusted mean difference in physically and mentally unhealthy days and days with activity limitation in people with and without arthritis. Results were adjusted for age, sex, dual Medicare/Medicaid eligibility, rural/urban commuting area, and Charlson Comorbidity Index. Asterisks in figure indicate significant differences in regression-adjusted means between the arthritis and nonarthritis groups at *P* < .001; error bars indicate 95% confidence intervals. Abbreviations: MUHD, mentally unhealthy days; PUHD, physically unhealthy days; UHD, unhealthy days. **Table:** 

After adjustment for age, sex, rural/urban commuting area, dual Medicaid/Medicare eligibility, and CCI, older adults with arthritis reported significantly more total unhealthy days than those without arthritis (arthritis: mean, 13.8 days, 95% confidence interval [CI], 13.1–14.4; no arthritis: mean 11.6 days, 95% CI, 11.0–12.3; *P* < .001). Similarly, individuals with arthritis reported significantly more physically and mentally unhealthy days and days with activity limitation than individuals without arthritis (Figure 1).

Condition	Adjusted Mean (95% Confidence Interval) No. of UHD		*P* Value
	Arthritis	No Arthritis	
PUHD	10.6 (10.0–11.2)	8.6 (8.0–9.2)	<.001
MUHD	6.1 (5.5–6.7)	5.6 (5.1–6.1)	<.001
Activity limitation	6.8 (6.3–7.3)	5.4 (4.8–6.0)	<.001

**Figure 2:**
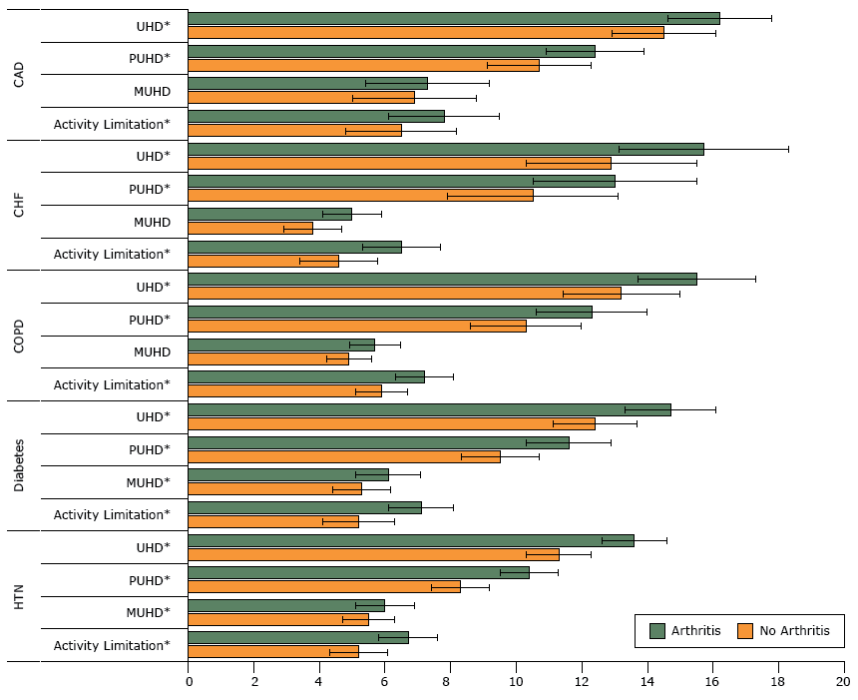
Adjusted mean difference in unhealthy days and days with activity limitation in people with a chronic condition, with and without arthritis. Results were adjusted for age, sex, dual Medicare/Medicaid eligibility, rural/urban commuting area, and Charlson Comorbidity Index. Asterisks in figure indicate significant differences in regression-adjusted means between the arthritis and nonarthritis groups at *P* < .001; error bars indicate 95% confidence intervals. Abbreviations: CAD, coronary artery disease; CHF, congestive heart failure; COPD, chronic obstructive pulmonary disease; HTN, hypertension; MUHD, mentally unhealthy days; PUHD, physically unhealthy days; UHD, unhealthy days. **Table:** 

In the study sample, 82% of individuals reported at least 1 of 5 common chronic conditions. Hypertension was the most prevalent (67%), followed by diabetes (30%), coronary artery disease (23%), chronic obstructive pulmonary disease (14%), and congestive heart failure (10%). Eighteen percent of the study population reported none of these 5 comorbidities. After adjustment, those with arthritis and either congestive heart failure, chronic obstructive pulmonary disease, diabetes, or hypertension reported significantly more total unhealthy days, physically and mentally unhealthy days, and days with activity limitation than those with the same chronic condition but without arthritis (Figure 2). For those with coronary artery disease, total and physically unhealthy days and days with activity limitation were significantly worse for those with arthritis, but mentally unhealthy days was not.

Condition	Adjusted Mean No. (95% Confidence Interval)		*P* Value
	No Arthritis	Arthritis	
**Hypertension**			
Activity limitation	5.2 (4.3–6.1)	6.7 (5.8–7.6)	<.001
MUHD	5.5 (4.6–6.4)	6.0 (5.2–6.8)	<.001
PUHD	8.3 (7.4–9.2)	10.4 (9.5–11.3)	<.001
UHD	11.3 (10.3–12.3)	13.6 (12.6–14.6)	<.001
**Diabetes**			
Activity limitation	5.2 (4.2–6.2)	7.1 (6.0–8.2)	<.001
MUHD	5.3 (4.3–6.3)	6.1 (5.2–7.0)	<.001
PUHD	9.5 (8.2–10.8)	11.6 (10.4–12.8)	<.001
UHD	12.4 (11.0–13.8)	14.7 (13.4–16.0)	<.001
**Chronic obstructive pulmonary disease**			
Activity limitation	5.9 (5.0–6.8)	7.2 (6.4–8.0)	<.001
MUHD	4.9 (4.1–5.7)	5.7 (5.0–6.4)	.01
PUHD	10.3 (8.6–12.0)	12.3 (10.6–14.0)	<.001
UHD	13.2 (11.4–15.0)	15.5 (13.7–17.3)	<.001
**Congestive heart failure**			
Activity limitation	4.6 (3.4–5.8)	6.5 (5.3–7.7)	<.001
MUHD	3.8 (2.9–4.7)	5.0 (4.1–5.9)	.003
PUHD	10.5 (8.0–13.0)	13.0 (10.4–15.6)	<.001
UHD	12.9 (10.3–15.5)	15.7 (13.1–18.3)	<.001
**Coronary artery disease**			
Activity limitation	6.5 (4.8–8.2)	7.8 (6.1–9.5)	<.001
MUHD	6.9 (5.0–8.8)	7.3 (5.4–9.2)	.15
PUHD	10.7 (9.2–12.2)	12.4 (10.8–14.0)	<.001
UHD	14.5 (12.9–16.1)	16.2 (14.6–17.8)	<.001

## Discussion

This study linked diagnoses from a large claims database to self-reported HRQoL to examine the effect of arthritis on HRQoL in adults with Medicare Advantage coverage. After adjusting for potential confounders, arthritis diagnosed through claims was associated with lower HRQoL and greater activity limitation, as has been reported using self-reported diagnosis of arthritis. Furthermore, this study expanded on previous studies, indicating an association between comorbid arthritis and even lower HRQoL among individuals with 5 common chronic conditions.

Our finding of more unhealthy days in older adults with a claims-based arthritis diagnosis than those without arthritis reinforces and builds on the published literature that examined this theme by using self-reported diagnoses (7–11). Compared with studies in populations including younger and older adults, we reported more mean total unhealthy days in those with arthritis (13.8 days vs 7.5–8.3 days) than in those without arthritis (11.6 days vs 3.7–4.5 days) but noted a smaller difference in total unhealthy days between individuals with and without arthritis (2.2 days in this study vs 3.8 days in other studies) (9,11). Similarly, studies by both Abell et al (8) and Shih and Simon (10), using self-reported arthritis in people aged 18 years or older, reported fewer physically unhealthy days (6.4 and 6.7 days with arthritis, 3.3 and 1.8 days without arthritis) and mentally unhealthy days (4.0 and 4.9 days with arthritis, 3.3 and 2.7 days without arthritis) (8,10) compared with our results. Such differences may be attributed to the larger comorbidity burden typical of older adults. However, a study using arthritis diagnosed through claims in an elderly Pennsylvania population found a similar number of reported physically and mentally unhealthy days (physically unhealthy days: 10.3–12.3 days with arthritis, 7.9 days without arthritis; mentally unhealthy days: 4.8–5.2 days with arthritis, 4.2 days without arthritis) (12). As with other studies, the number of physically unhealthy days was higher than that of mentally unhealthy days (8,10,12). The comparison of the regression-adjusted means of the HRQoL measures accounts for the correlation observed in these measures between those with arthritis and those without arthritis; as a result, the *P* values may seem more extreme than one would expect from comparing confidence intervals.

To our knowledge, no other study has focused on determining the effect of arthritis on Healthy Days in the presence of other chronic conditions. The prevalent and debilitating comorbidities in our study were each consistently associated with more than 10 physically and 5 mentally unhealthy days. Our results showed significant increases in both physically and mentally unhealthy days for individuals with comorbid hypertension, diabetes, chronic obstructive pulmonary disease, and congestive heart failure in addition to their arthritis, compared with individuals with the same comorbidities in the absence of arthritis. Interestingly, only reported physically unhealthy days were significantly higher in individuals with coronary artery disease and arthritis compared with those with the comorbidity but without arthritis. Data from other studies support our results. Furner et al, who sought to determine the correlates of poor HRQoL in adults with self-reported arthritis, found a substantial increase in total unhealthy days for those reporting hypertension (12.2 days with arthritis, 6.6 days without arthritis) and diabetes (15.4 days with arthritis, 8.2 days without arthritis) (11). Another study of adults with arthritis aged 45 years or older, 81% of whom had at least 1 comorbidity, reported 7.7 physically and 4.4 mentally unhealthy days (7). Differences in population age distribution and comorbidities, which were not specified in this comparison study, may explain the lower reported unhealthy days compared with our study.

Although this study benefited from a large number of Healthy Days responses that could be linked to a claims-based diagnoses, certain study limitations exist. First, the Healthy Days survey data are subject to response and recall bias. Although weighting was used to address potential selection bias due to survey nonresponse, some residual bias may remain. Second, administrative claims data have inherent limitations, such as coding errors and missing data. Third, potential confounders that may have affected unhealthy days (such as race/ethnicity, socioeconomic characteristics, employment and marital status, arthritis severity, and years of arthritis) were not available in claims data and thus were not adjusted for in the analysis. However, limitations of survey bias, claims errors, and unmeasured confounders should have affected the arthritis and no arthritis groups equally and thus have little effect on the internal validity of our findings. Fourth, the analysis was weighted using the survey sample weights, making our results generalizable to the Humana Medicare Advantage population aged 65 years or older with 1 year of continuous enrollment; however, our results may not be generalizable to a younger, commercially insured population residing in geographies not well represented in our population. Finally, affecting Healthy Days in people with chronic arthritis may be difficult, because treatment may not be as effective in chronic arthritis as in more recently diagnosed arthritis (29).

Arthritis is an important determinant of HRQoL, and for people with other chronic conditions, comorbid arthritis is associated with further reduced HRQoL. Given the high prevalence of chronic comorbid conditions and arthritis in older adults, improving HRQoL depends upon managing both chronic conditions and any accompanying arthritis. Engaging in regular physical activity can reduce the disability associated with arthritis and the reported number of unhealthy days; however, adherence to physical activity recommendations is low among individuals with arthritis (7,8). Our findings support this fact, because we found that the mean number of days with activity limitation was higher for people with arthritis than for those without arthritis, overall and for each chronic condition. Current strategies to improve physical activity adherence include primary care physician discussions about ways to be more active (eg, walking, biking, swimming) and community programs for safely increasing physical activity among those who worry about arthritis-specific barriers (eg, pain, worsening arthritis). Additional research and strategies on effective ways to assist people, especially older adults, in meeting physical activity recommendations is warranted. Self-management strategies, such as the Chronic Disease Self-Management Programs, help those with both arthritis and common chronic conditions (heart disease, diabetes, and obesity). Interventions to address social determinants of health, such as income level, housing, and education, are also important opportunities for reducing disability and improving quality of life in this population (30,31).

## Appendix A. Survey and Study Populations and B. Diagnosis Codes Used to Identify Chronic Conditions of Interest

This document is available for download as a Microsoft Word file.
