# Supplementary material for: Comorbid Arthritis Is Associated With Lower Health-Related Quality of Life in Older Adults With Other Chronic Conditions, United States, 2013–2014
Source: Prev Chronic Dis. 2017 Jul 27;14:E60. doi: 10.5888/pcd14.160495 (PMC5542545; doi:10.5888/pcd14.160495)
Supplement: Supplementary file 1 [file 16_0495Appendix.docx]

Appendix A. Survey and study populations


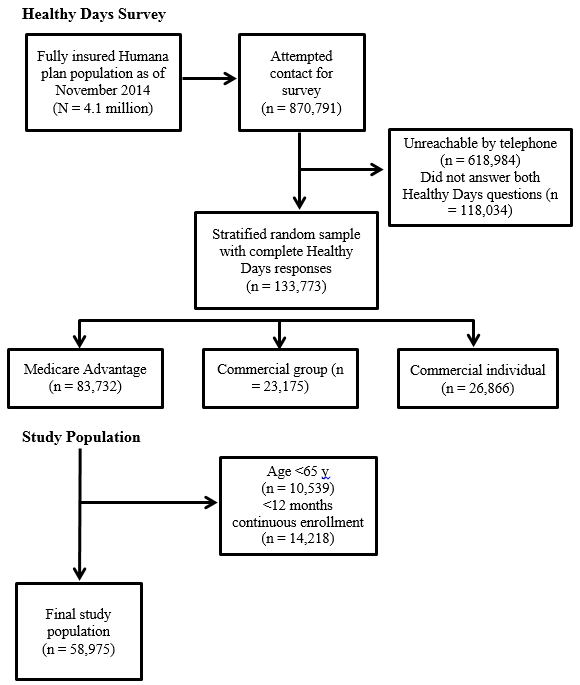


Appendix B. Diagnosis codes used to identify chronic conditions of interest

| **Chronic Condition** | **ICD-9 Codes** |
| --- | --- |
| Chronic obstructive pulmonary disease | 491.0-491.22, 491.8, 491.9, 492.0, 492.8, 496, 518.1, 518.2 |
| Coronary artery disease | 410.0-414.9, 429.5, 429.6, V4581-V4582 |
| Congestive heart failure | 398.91, 402.01, 402.11, 402.91, 404.01, 404.11, 404.91, 415.0, 428.0-428.43, 428.9 |
| Diabetes | V5867, 249.0-250.9 |
| Hypertension | 401.0, 401.1, 401.9, 997.91 |

ICD-9, International Classification of Diseases, 9^th^ Revision
